# The 3′ Untranslated Region of a Plant Viral RNA Directs Efficient Cap-Independent Translation in Plant and Mammalian Systems

**DOI:** 10.3390/pathogens8010028

**Published:** 2019-02-28

**Authors:** Jelena J. Kraft, Mariko S. Peterson, Sung Ki Cho, Zhaohui Wang, Alice Hui, Aurélie M. Rakotondrafara, Krzysztof Treder, Cathy L. Miller, W. Allen Miller

**Affiliations:** 1 Department of Plant Pathology and Microbiology, Iowa State University, Ames, IA 50011, USA; jelenajk@iastate.edu (J.J.K.); skcho011@gmail.com (S.K.C.); zhaohuiw@wustl.edu (Z.W.); 2 Department of Genetics, Development and Cell Biology, Iowa State University, Ames, IA 50011, USA; 3 Department of Biochemistry, Biophysics and Molecular Biology, Iowa State University, Ames, IA 50011, USA; mariko.peterson@emory.edu; 4 Yerkes National Primate Research Center, Emory Vaccine Center 2009, 954 Gatewood Rd NE, Atlanta, GA 30329, USA; 5 Dura-Line, 1355 Carden Farm Dr., Clinton, TN 37716, USA; 6 Department of Orthopaedic Surgery, Washington University School of Medicine, St. Louis, MO 63110, USA; 7 Interdepartmental Plant Biology Program, Iowa State University, Ames, IA 50011, USA; ahuh41@gmail.com; 8 Department of Plant Pathology, University of Wisconsin, Madison, WI 53706, USA; rakotondrafa@wisc.edu; 9 Laboratory of Molecular Diagnostic and Biochemistry, Bonin Research Center, Plant Breeding and Acclimatization Institute–National Research Institute, 76-009 Bonin, Poland; k.treder@ihar.edu.pl; 10 Department of Veterinary Microbiology and Preventive Medicine, College of Veterinary Medicine, Iowa State University, Ames, IA 50011, USA; clm@iastate.edu

**Keywords:** 5′ cap, translation initiation, RNA binding protein, Tombusviridae, plant virus, 3′ untranslated region, pseudoknot, cap-independent translation

## Abstract

Many plant viral RNA genomes lack a 5′ cap, and instead are translated via a cap-independent translation element (CITE) in the 3′ untranslated region (UTR). The panicum mosaic virus-like CITE (PTE), found in many plant viral RNAs, binds and requires the cap-binding translation initiation factor eIF4E to facilitate translation. eIF4E is structurally conserved between plants and animals, so we tested cap-independent translation efficiency of PTEs of nine plant viruses in plant and mammalian systems. The PTE from thin paspalum asymptomatic virus (TPAV) facilitated efficient cap-independent translation in wheat germ extract, rabbit reticulocyte lysate, HeLa cell lysate, and in oat and mammalian (BHK) cells. Human eIF4E bound the TPAV PTE but not a PTE that did not stimulate cap-independent translation in mammalian extracts or cells. Selective 2′-hydroxyl acylation analyzed by primer extension (SHAPE) footprinting revealed that both human and wheat eIF4E protected the conserved guanosine (G)-rich domain in the TPAV PTE pseudoknot. The central G plays a key role, as it was found to be required for translation and protection from SHAPE modification by eIF4E. These results provide insight on how plant viruses gain access to the host’s translational machinery, an essential step in infection, and raise the possibility that similar PTE-like mechanisms may exist in mRNAs of mammals or their viruses.

## 1. Introduction

All plant and vertebrate viruses are parasites of the host’s translational machinery because they do not encode any of its components [1]. Thus, a key host-pathogen interaction in establishing infection is that of viral mRNA with the host’s translation factors and the ribosome. The genomes of positive strand RNA viruses which, by definition, also serve as mRNA, do not replicate in the nucleus where capping of host mRNAs takes place. Thus, they either encode capping enzymes [2], which is a genetic burden for most RNA viruses, or their RNA remains uncapped. For uncapped viral RNAs to translate efficiently, they harbor a structure in their untranslated region (UTR) that recruits translational machinery in the absence of a 5′ cap. In animal viruses, such a structure is either a genome-linked protein (VPg) covalently attached to the 5′ end in the case of noroviruses [3], or more often, an internal ribosome entry site (IRES) in the viral RNA. An IRES is a highly structured RNA element of several hundred bases that commandeers host proteins called IRES trans-acting factors (ITAFs) to recruit the ribosome to the IRES, without requirement for scanning from the 5′ end [4,5,6]. In plant viruses, IRESes have been reported, but, with one apparent exception [7], they are smaller, less structured, and not as efficient as IRESes in animal virus RNAs [8,9,10,11]. Instead of an IRES, many plant viruses contain a sequence in their 3′ UTR, which allows efficient cap-independent translation initiation via ribosome scanning from the 5′ end of the genome [12,13,14]. These 3′ cap-independent translation elements (3′ CITEs) bind a surface of translation initiation factor heterodimer eIF4F with high affinity and, in most cases, are thought to deliver this and possibly other factors and the 40S ribosomal subunit, to the 5′ end via long-distance base pairing between the 3′ and 5′ UTR [15,16,17,18,19,20,21].

3′ CITEs fall into at least seven categories, each with an entirely different structure [9,14]. These include (i) the translation enhancer domain (TED) of satellite tobacco necrosis virus, consisting of a stem-loop with multiple bulges, [22,23], (ii) the barley yellow dwarf virus (BYDV)-like 3’ CITE (BTE) consisting of two to five stem-loops branching from a stem helix at a single junction, with one stem-loop highly conserved in sequence [24,25,26], (iii) the I-shaped domain of melon necrotic spot virus [18,27], (iv) Y-shaped domain of carnation Italian ringspot virus [17], (v) the tRNA-like T-shaped structure of turnip crinkle virus (TCV) [28,29,30], (vi) the panicum mosaic virus-like CITE (PTE) [15,31,32], and (vii) a pair of stem-loops forming a dumbell-shape in the Xinjiang isolate (but absent in European isolates) of cucurbit aphid-borne yellows virus (CABYV-X) also found in a resistance-breaking strain of Melon necrotic spot virus (MNSV) [33]. With the exception of CABYV-X, all of the above viruses are in the *Tombusviridae* family or the closely related *Luteovirus* genus of the *Luteoviridae* family [9,14].

In plants, the key translation initiation factor complex, eIF4F, consists of two subunits, the cap-binding protein eIF4E, and the scaffolding protein eIF4G, which recruits other factors, including eIF4A and eIF4B, which together have helicase activity that aid in ribosome scanning [34]. The BTE binds the eIF4G subunit of eIF4F [16] and does not require eIF4E, eIF4A, or eIF4B, although these factors enhance the interaction of eIF4G with the BTE [16,19]. The TED [35] and Y-shaped structures [17] appear to bind the eIF4F heterodimer only, with little affinity for either subunit alone, while the TCV T-shaped structure binds directly to the 60S ribosomal subunit [36]. The I-shaped structure [37] and the PTE [15] bind the eIF4E subunit of eIF4F. The I-shaped structure requires presence of at least a portion of eIF4G bound to eIF4E, whereas the PTE binds eIF4E alone [15]. This is remarkable in that the presence of the m^7^G on the cap-structure on mRNA was thought to be required for eIF4E to bind RNA [38]. All 3’ CITEs require eIF4G for function and bind with higher affinity to eIF4F than to the individual subunits alone. Plants, but not animals, contain two isoforms of the subunits of eIF4F: eIF4F, which consists of eIF4E plus eIF4G, and eIFiso4F, which consists of eIFiso4E plus eIFiso4G [39]. eIFiso4E has about 50% sequence identity to eIF4E, while eIF4isoG is about 60% of the size of eIF4G with major deletions in domains of unknown function [40]. eIF4F stimulates cap-dependent translation, and BTE- [16] and PTE- [15] mediated translation more efficiently than eIFiso4F. Thus, this paper focuses only on interactions of PTEs with eIF4E.

**Figure 1:**
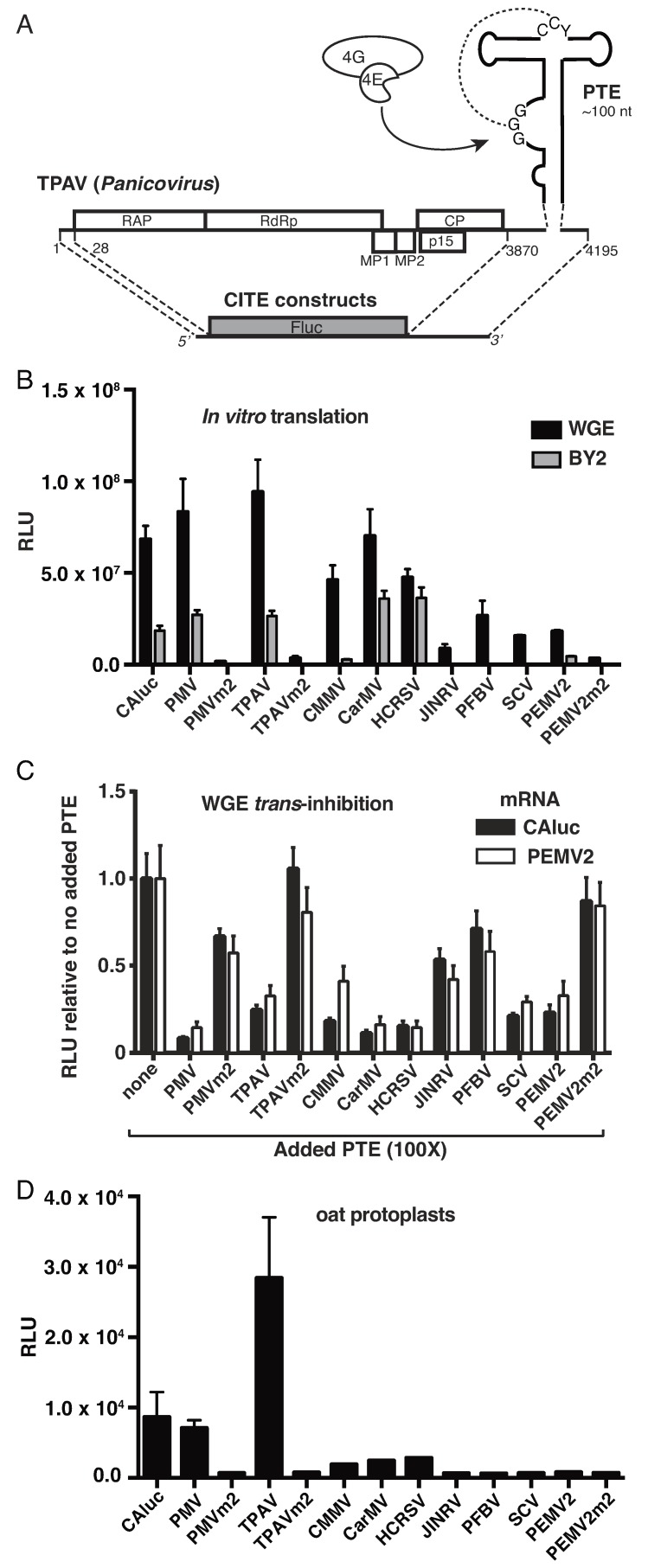
Translation in plant systems. (**A**) Genome organization of thin paspalum asymptomatic virus (TPAV) as a representative of the *Tombusviridae,* and luciferase reporter construct. Secondary structure and position of the panicum mosaic virus-like CITE (PTE), which binds the eIF4E subunit of eIF4F (arrow) is shown in the 3′ untranslated region (UTR). Curved dashed line indicates potential pseudoknot base pairing between C domain (CCY, Y = pyrimidine), and G domain (GGG). TPAV open reading frames (ORFs), indicated by boxes are named as in Ref. [41]. For each viral luciferase construct tested in panels B–D, the complete 5′ and 3′ UTR of each virus flank the firefly luciferase ORF (cap-independent translation element (CITE constructs). (**B**) Relative luciferase activity produced in wheat germ extract or tobacco BY2 cell extract by translation of 20 nM uncapped reporter mRNAs (CITE constructs, panel A) containing the 5′ and 3′ UTR of the indicated virus. m2 indicates CITE construct with a CC→AA mutation in the C-domain of the PTE. (**C**) Translation of 2 nM uncapped pea enation mosaic virus 2 (PEMV2) CITE construct (open bars) or capped, non-viral (CAluc, black bars) mRNA in wheat germ extract in the presence of 200 nM of the indicated PTEs. (**D**) Relative luciferase activity in oat protoplasts produced by indicated uncapped viral CITE constructs. The data shown are averages of triplicates from two experiments and error bars represent standard error.

Previously, secondary structural analysis of PTEs of nine different tombusvirids revealed that they have little sequence conservation, and vary in many ways, but all form a roughly T-shaped structure with branching stem-loops in which a (usually) C-rich joining sequence (4–6 nt) at the branch-point of the stem-loops has potential to base pair to a G-rich bulge (~8–11 nt) in the main stem, forming a pseudoknot (Figure 1A and Supplementary Figure S1) [32]. A diagnostic feature of all PTEs is that one G in the G-rich bulge can be hypermodified by SHAPE reagents in a magnesium-dependent manner [32]. This G is not hypermodified in the absence of magnesium ion, or if the C-rich sequence is mutated to disrupt potential base pairing to the G-rich bulge. These mutations also inactivate the PTE [15]. Correspondingly, eIF4E protects bases in the C-rich and G-rich regions from modification by SHAPE reagents, indicating that this pseudoknot base-pairing is the likely eIF4E binding site [32].

To understand the structural basis for high affinity binding of eIF4E to the PTE in the absence of a 5′ cap, we undertook a comparative approach. eIF4E varies substantially in sequence, but only slightly in three-dimensional structure across kingdoms. Thus, we compared the efficiency of nine different PTEs in facilitating cap-independent translation in mammalian cells and cell lysates, and the ability of human eIF4E to bind two of these PTEs. We found a wide range in efficiency of translation facilitated by different PTEs, ranging from background levels, to half that conferred by one of the most active mammalian virus IRESes known. The efficiency of translation stimulation correlated with binding affinity of the PTE to eIF4E. To our knowledge, this work reveals the first 3′ CITE that functions in mammalian cells. This PTE may be a useful tool for engineered gene expression in mammalian systems, and it opens the possibility that such elements may exist in mammalian viral RNAs.

## 2. Results

### 2.1. Thin Paspalum Asymptomatic Virus (TPAV) Has the Most Powerful PTE

PTEs vary substantially in sequence, length of branching stem-loops, and predicted stability of pseudoknot base pairing (Supplementary Figure S1) [32]. To determine how these structural variations affect translation initiation efficiency, we compared the cap-independent translation stimulation activities of nine different PTEs in plant translation systems. We employed a reporter system which contained the 5′ and 3′ UTR of each virus flanking the firefly luciferase ORF (Figure 1A). Under non-saturating mRNA concentrations, at short time points at which the rate of translation is most rapid and sensitive to differences in efficiency, we observed wide variation in efficiencies of translation mediated by the different PTEs in monocot (wheat germ extract) and dicot (tobacco BY2 cell lysate) systems. The UTRs containing PTEs of panicoviruses *Panicum mosaic virus* (PMV) and *Thin paspalum asymptomatic virus* (TPAV), alphacarmovirus *Carnation mottle virus* (CarMV) and betacarmovirus *Hibiscus chlorotic ringspot virus* (HCRSV) yielded as much luciferase activity from uncapped transcript as a capped, polyadenylated transcript (CAluc) containing nonviral UTRs (Figure 1B). UTRs of *Japanese iris necrotic ringspot virus* (JINRV, *Betacarmovirus*), *Pelargonium flower break virus* (PFBV, *Alphacarmovirus*), *Saguaro cactus virus* (SCV, *Alphacarmovirus*) and *Pea enation mosaic virus 2* (PEMV2, *Umbravirus*), stimulated translation to levels several fold below those of the most active PTEs. The translation activity in the BY2 lysates was lower than that of the WGE, but the performance of the PTEs relative to each other was similar in both systems. As a negative control we tested mutation m2, which disrupts the pseudoknot by altering two conserved cytosine residues in the “C-rich domain” of the PTE to adenosines, and was shown previously to abolish translation enhancement activity of the PEMV2 PTE [15]. Here we found that the m2 mutation abolished translation enhancement of the most active PTEs: PMV and TPAV, as well as that of PEMV2 (PMVm2, TPAVm2, and PEMV2m2, Figure 1B).

Previously it was shown that addition of a 3′ CITE to a translation reaction in *trans* inhibits translation of an mRNA containing a 5′ cap or a 3′ CITE in *cis*, to a degree that reflects the 3′ CITE’s efficiency in stimulating translation in *cis* [16,18,24,42]. Thus, we tested the relative efficiencies of each PTE to inhibit translation of a capped, polyadenylated mRNA (CAluc) or an uncapped mRNA containing the PEMV RNA2 5′ and 3′ UTRs. The PEMV2 3′ UTR includes the PTE, and two other reported translation enhancers: a tRNA-shaped structure (TSS) that binds the 60S ribosomal subunit, and a kissing stem-loop TSS (kl-TSS) [43,44,45]. In general, the PTEs that conferred greatest translation in *cis* (Figure 1B), were the strongest inhibitors of translation in *trans*, with possible exceptions of SCV and PEMV2 PTEs which stimulated weakly in cis but inhibited as strongly as TPAV in *trans* (Figure 1C). Differences between *trans*-inhibition and *cis*-stimulation may be due to the different sequence context. The PTE used in *trans* may fold differently than in the context of the reporter mRNA with the full 3′ UTR in *cis*. The PEMV2 PTE may function incompletely in *cis* owing to incomplete sequence at the 5′ end reported to base pair to the 3′ UTR [45]. Most importantly, the three PTEs containing the m2 mutation, PMVm2, TPAVm2, and PEMV2m2, inhibited translation in *trans* very weakly or not at all (Figure 1C). In all cases, the level of inhibition of capped nonviral mRNA was similar to that of uncapped RNA containing the PEMV RNA2 PTE, TSS and kl-TSS structures in its 3′ UTR, consistent with the possibility that the *trans*-inhibition acts by the PTEs binding and sequestering eIF4E.

In vivo (oat protoplasts), the differences in translation efficiency of the reporter constructs were much more striking than in cell-free extracts. Translation mediated by the PMV PTE was similar to that of capped, polyadenylated mRNA, while the TPAV PTE yielded three times as much translation activity (Figure 1D). In contrast, the weakest PTEs, as measured in WGE, gave levels of translation in protoplasts that were barely above that of the negative control m2 mutants (Figure 1D).

### 2.2. The TPAV PTE Confers Efficient Cap-Independent Translation in Mammalian Extracts and Cells

**Figure 2:**
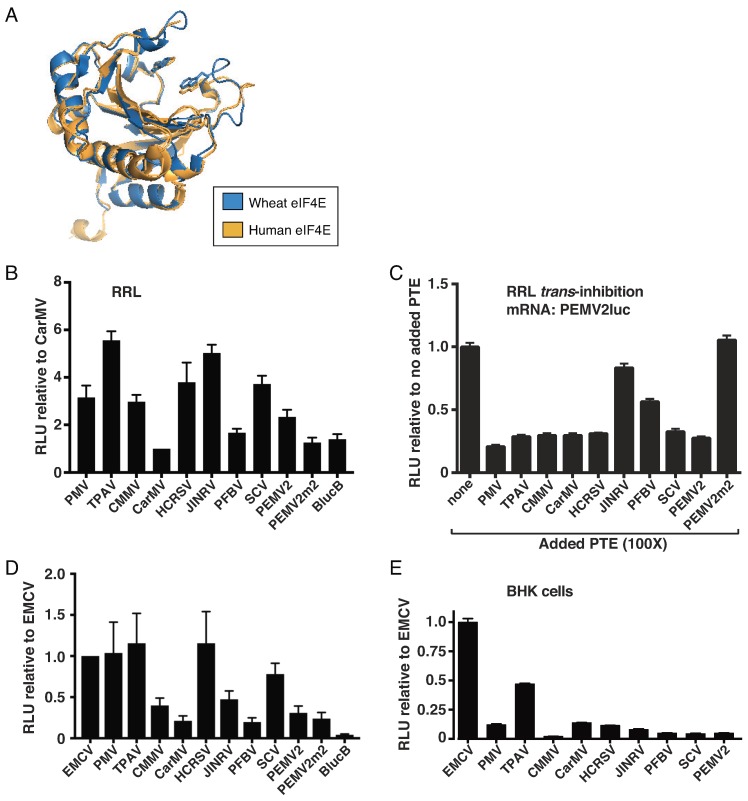
PTE-mediated cap-independent translation in mammalian systems. (**A**) Overlay of wheat (blue) and human (amber) eIF4E structures (Swiss PDB Viewer and PovRay, accession numbers 2IDV and 1IPC, respectively), showing tryptophan residues that sandwich around the m^7^GTP cap. (**B**) *Cis*-translation results of uncapped viral reporter RNAs (CITE constructs, Figure 1A) in rabbit reticulocyte lysate (RRL), with relative light units (RLU) shown relative to those produced by the CarMV CITE construct. BlucB is a luciferase reporter containing the 5′ and 3′ UTRs of barley yellow dwarf virus (BYDV), which harbors a BYDV-like translation element (BTE) rather than a PTE in its 3′ UTR. (**C**) *Trans*-inhibition of translation of uncapped PEMV2 CITE construct mRNAs by PTEs as in Figure 1C, but in RRL. (**D**) *Cis*-translation of uncapped viral CITE construct RNAs in HeLa cell lysate. RLUs are relative to those obtained from firefly luciferase RNA containing the EMCV IRES in its 5′ UTR. (**E**) *Cis*-translation results of viral CITE construct RNAs relative to EMCV reporter in DNA-transfected BHK-T7 cells. Plasmids were linearized with Sma I as for in vitro transcription of RNAs used in in vitro assays. The data shown are averages of triplicates from at least two experiments, with error bars representing standard error.

The PTE binds eIF4E to facilitate cap-independent translation [15]. The structure of eIF4E is highly conserved across kingdoms, as demonstrated by structural overlay with an RMS value of 0.86 Å (Figure 2A). Thus, we tested whether the PTEs from diverse tombusvirids could support translation in mammalian systems, including rabbit reticulocyte lysate (RRL), HeLa cell lysate, and in BHK cells expressing T7 polymerase. Long ago, CarMV RNA was shown to translate in RRL [46], so, we used the reporter with the CarMV PTE for comparison. We found that most PTEs facilitated more efficient translation (Figure 2B). In fact, the CarMV PTE translated no better than the BYDV BTE (BlucB). The BTE (i) is structurally unrelated to PTEs, (ii) binds eIF4G, (iii) is nearly eIF4E-independent [16], (iv) is highly efficient in plant systems [47], but (v) was shown previously not to stimulate efficient translation in RRL [48]. TPAV, PMV, and HCRSV PTEs strongly stimulated translation in *cis* in both RRL and HeLa lysate, and inhibited translation in trans in RRL (Figure 2C), while other PTEs were more variable between the two systems. We used the encephalomyocarditis virus (EMCV) IRES as a positive control in HeLa lysates and in baby hamster kidney (BHK) cells, because it is a strong IRES in mammalian cells [49,50]. However, the EMCV IRES does not stimulate translation in RRL in our conditions (i.e., in the absence of viral 2A protease cleavage of eIF4G) [51].

To test the activity of the PTEs in vivo, we used BHK cells that constitutively express bacteriophage T7 RNA polymerase [52]. These cells were transfected with the same linearized plasmids used for in vitro transcription of the above luciferase reporter mRNAs containing the viral UTRs. The uncapped mRNA was then transcribed from these plasmids by the intracellular T7 polymerase in vivo. As expected, the EMCV IRES yielded high levels of luciferase activity (Figure 2E). Notably, the reporter containing the TPAV PTE yielded half as much luciferase as the powerful EMCV IRES, indicating that the TPAV PTE facilitates quite efficient cap-independent translation in mammalian cells. In contrast, the other PTEs gave substantially lower levels of translation. In summary, while there was variability of performance by some PTEs in the different translation systems, the TPAV PTE consistently yielded high levels of translation in all plant and animal systems, especially in living cells.

### 2.3. The TPAV PTE Binds Human eIF4E

**Figure 3:**
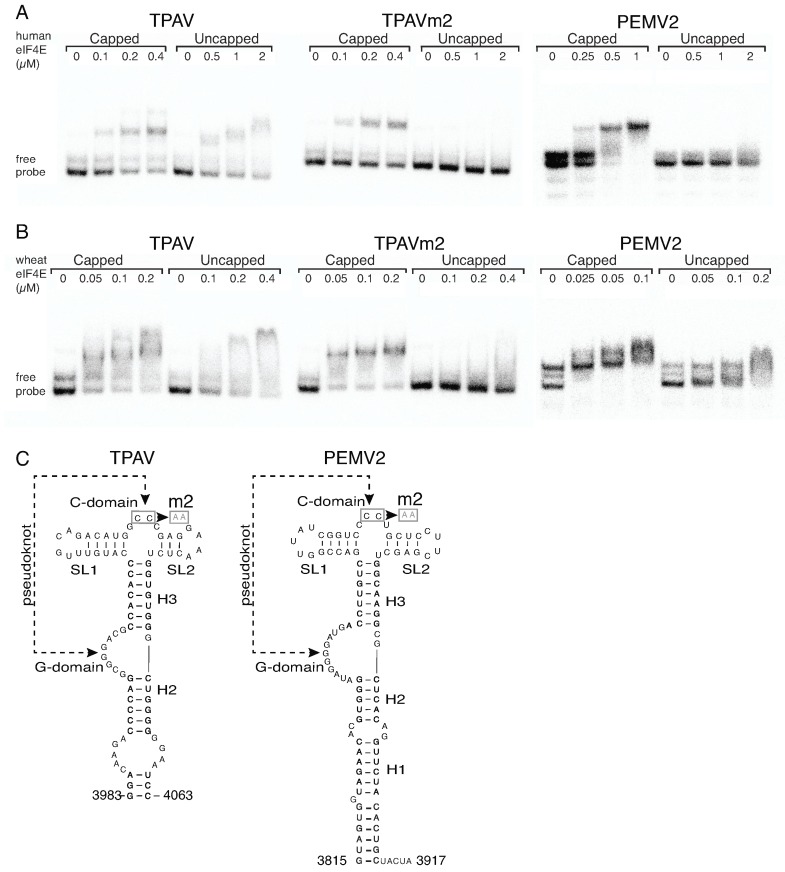
Electrophoretic mobility shift assay (EMSA) of eIF4E binding to the PTE. Twenty femtomoles of ^32^P-labeled capped or uncapped PTE RNAs and human (**A**) or wheat (**B**) eIF4Es at the indicated concentrations were used in each reaction. Panel (**C**) shows secondary structures and m2 mutations of the TPAV and PEMV2 PTEs. See Materials and Methods for details.

Previously we found with mutant PTEs that the efficiency of the PTE in translation correlated generally with its ability to bind eIF4E [15]. Thus, because the TPAV PTE is the most efficient in mammalian systems, we hypothesize that it binds mammalian eIF4E with higher affinity than do other PTEs. To test this hypothesis, we performed electrophoretic mobility shift assays (EMSA) to observe binding of human or wheat eIF4E to radiolabeled TPAV PTE, nonfunctional mutant TPAVm2 PTE, or the PEMV2 PTE. As positive controls for eIF4E function, we used capped versions of all PTEs tested. The mobilities of all of the capped RNAs were clearly shifted by added human or wheat eIF4E (Figure 3). Mobility of uncapped TPAV PTE shifted in the presence of ≥0.5 µM human eIF4E, whereas nonfunctional mutant TPAVm2 and PEMV2 PTEs were not shifted by 2 µM human eIF4E. For comparison, both uncapped TPAV PTE and uncapped PEMV2 PTEs showed substantial shifting by wheat eIF4E. PEMV2 PTE migrated as three bands in the absence of protein, indicating multiple conformations, but it is clear that in the presence of 0.2 µM wheat eIF4E, that the mobility is slower than in the absence of protein (Figure 3B, far right gel). Estimated dissociation constants were calculated from multiple EMSAs as the amount of eIF4E needed to shift one-half of the RNA. The TPAV PTE bound human eIF4E with about three times the affinity as PEMV2 PTE and about 7.5-fold the affinity as the TPAVm2 mutant (Table 1).

An interesting feature we observed consistently in all replicates of the EMSA experiments, was that the mobility of functional PTEs was increasingly reduced in the presence of increasing concentrations of eIF4E (human or wheat) (Figure 3A, TPAV; Figure 3B, TPAV, PEMV2). In contrast, the shifted mobilities of the capped, nonfunctional PTEs remained constant, and simply became more intense with increasing concentrations of eIF4E (Figure 3A, TPAVm2, PEMV2; Figure 3B, TPAVm2). This indicates that the molecular interactions of eIF4E binding the uncapped PTE may somehow differ from those involved in binding the 5′ cap. We conclude from these EMSA experiments, that (i) wheat eIF4E binds with higher affinity than human eIF4E to all PTEs and capped RNAs, (ii) human and wheat eIF4E bind with high affinity only to those uncapped PTEs that function in mammalian or plant extracts respectively, and (iii) eIF4E (human or wheat) binding to the uncapped PTE may involve different interaction(s) than to capped RNA, or may alter the RNA structure differently.

### 2.4. The G-Rich Bulge (G-Domain) of the PTE Is Involved in eIF4E Binding

**Figure 4:**
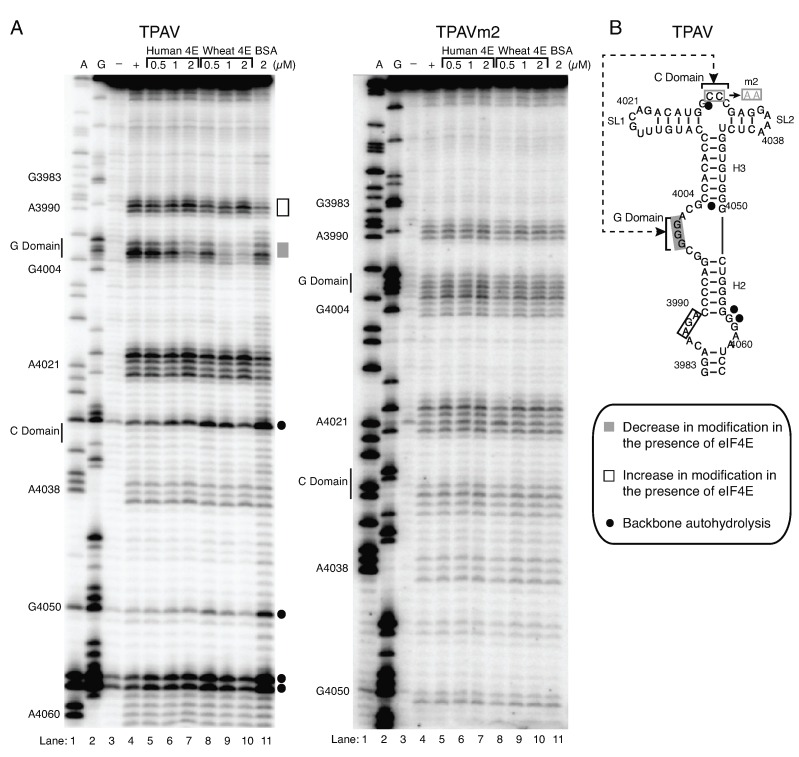
(**A**) TPAV and TPAVm2 PTE modification and cleavage structure probing patterns generated using the SHAPE reagent benzoyl cyanide (BzCN) in the absence of protein (+ lane) or presence of indicated proteins (lanes 5–11). A and G are dideoxy sequencing lanes, with positions of selected bases indicated at left. Unmodified RNA (− lane) shows background RNA hydrolysis. Gray and white bars to the right show regions with increased or decreased, respectively, modification by BzCN in the presence of eIF4E. Filled circles at right denote regions of autohydrolysis in the absence of BzCN. (**B**) Positions of human eIF4E-induced changes in SHAPE sensitivity mapped onto the TPAV PTE secondary structure. Double-headed arrow indicates predicted pseudoknot base pairing between the C and G domains, which is disrupted by the CC to AA changes in mutant m2 (boxed in gray).

To further compare the interaction of wheat and human eIF4E with the TPAV PTE, we used SHAPE modification of the TPAV PTE in the presence of increasing quantities of eIF4E. Neither wheat nor human eIF4E had much effect on the overall structure, and instead revealed changes at discrete sites. In particular, the G-rich bulge (G-domain) became much less susceptible to modification by benzoyl cyanide in the presence of eIF4E from either kingdom (see Figure 4 and Supplementary Figure S2). This modification was inhibited more strongly by wheat than human eIF4E, as may be expected based on the greater binding affinity of wheat than human eIF4E to the TPAV PTE (Figure 3, Table 1). A possible increase in modification in the presence of either eIF4E was seen around base 3990 at the base of the PTE. No other changes in the modification patterns were seen. This differs from a previous study in which a slight decrease in modification of the C-domain was seen [32], although in all studies, the C-domain is only weakly modified even in the absence of eIF4E. Importantly, the same concentrations of human and wheat eIF4E had no effect on modification of nonfunctional TPAVm2 PTE (Figure 4), consistent with the lack of binding in the EMSA (Figure 3). We conclude that the structure of the G domain becomes more rigid and thus less modified due to eIF4E binding to the functional TPAV PTE and that this is the likely binding site for eIF4E of both wheat and human.

**Figure 5:**
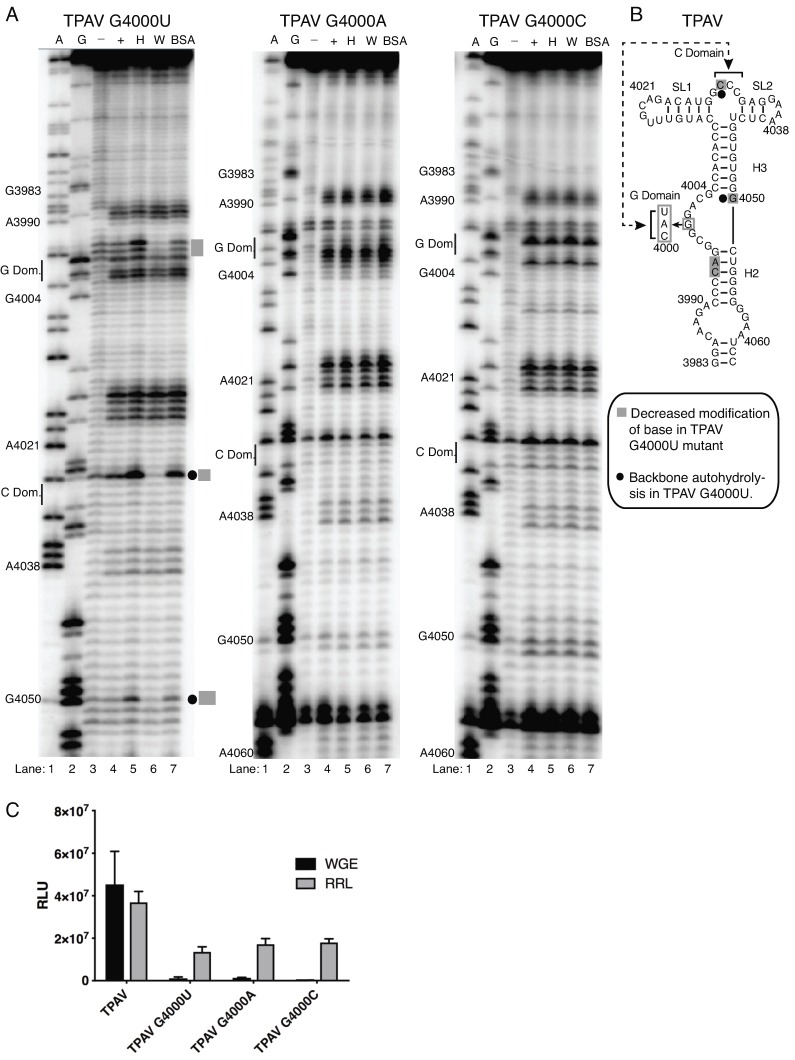
Effect of G4000 mutations in TPAV PTE on structure and translation efficiency. (**A**) BzCN footprinting of the TPAV PTE with indicated mutation at the hypermodified G4000 residue in the presence of human eIF4E (H), wheat eIF4E (W) or bovine serum albumin (BSA). All proteins were added to final concentration of 2 μM. A and G are dideoxy sequencing lanes showing positions of A and G residues, respectively, in the RNA template. Unmodified RNA (−) shows background RNA hydrolysis, (+) indicates BzN modification in the absence of added protein. Gray bars to the right show regions protected by wheat eIF4E from BzCN modification (G4000U mutant only). Small filled circles at right denote regions of autohydrolysis in the absence of BzCN. (**B**) TPAV secondary structure showing hypermodified G4000 mutants and sequences protected by wheat eIF4E in the G4000U point mutant from chemical modification by BzCN. (**C**) Relative luciferase activity produced in wheat germ extract (WGE) and rabbit reticulocyte lysate (RRL) by the translation of indicated mutant TPAV viral CITE construct reporter mRNAs.

Previous structural studies of the PTE-eIF4E interaction from other viruses indicated that the G-domain and in particular middle G4000 nucleotide is involved in the binding to the wheat eIF4E likely in the cap-binding pocket [32]. To determine the importance of the G domain in the TPAV PTE, we investigated the effects of altering the middle guanosine (G4000) of the GGG tract to each of the other three bases on eIF4E binding and on translation activity. In all three mutants tested we failed to observe the protection at this position from the benzoyl cyanide modification in the presence of either human or wheat eIF4E, suggesting that the G nucleotide and/or the local structure of this domain is critical for binding to eIF4E. Indeed, we did observe the alteration in the structure of G-domain by the point mutations although the rest of the PTE secondary structure of the three mutants was similar to the wild type TPAV PTE structure (+ lane, Figure 4 and Figure 5).

We did however observe that the presence of wheat but not human eIF4E conferred protection at bases C3994, A3995 in the G4000U mutant that was absent in the wild type TPAV PTE and all other mutants (Figure 4 and Figure 5). Additionally, wheat eIF4E reduced the backbone hydrolysis in the G4000U mutant at bases G4050 and C4029 located in the C and H3 domains respectively (Figure 5) that was not apparent in wild type TPAV PTE (Figure 4 and Figure 5), suggesting that eIF4E binding is altered in this mutant relative to its binding to the wild type TPAV PTE. The absence of the productive eIF4E binding (wheat or human) to all three G-domain mutants in the SHAPE analysis correlates with the lack of ability of these mutants to stimulate translation in wheat germ and RRL. All three mutations essentially abolished translation stimulation in wheat germ extract, and reduced translation in RRL by over 50% (Figure 5C). This higher level of translation of mutants in RRL, relative to wheat germ extract, reflects the lesser cap-dependence in RRLs compared to wheat germ extract [48], and the fact that the stimulation level of translation by the wild type TPAV PTE is greater in wheat germ extract than in RRLs. Thus, the ability of the PTE to facilitate translation correlates with ability of eIF4E to interact with the G-domain, and specifically the middle G, which is required for cap-independent translation. Importantly, in all cases where eIF4E (wheat or human) had no effect on SHAPE probing profile, the PTE did not facilitate efficient translation. Future high-resolution structural analysis would be beneficial to discern the specific mode of interaction between the G-domain of TPAV PTE and eIF4E.

## 3. Discussion

### 3.1. Interaction of Mammalian eIF4E with TPAV PTE

SHAPE probing here and previously revealed a highly modified G residue in the G-rich bulge of all PTEs. Despite its high modification, this G is predicted to interact with the C-rich region at the three-way helical junction in a possible pseudoknot interaction (Figure 4B) [32]. In the presence of eIF4E, the G-domain bulge becomes less sensitive to SHAPE probing, indicating reduced flexibility in structure and possible binding at this site by eIF4E. This led us to propose that the highly modified G protrudes into the cap-binding pocket of eIF4E, and that amino acids surrounding the pocket interact with nucleotides around the G-rich bulge to strengthen the interaction [32]. The fact that altering the highly modified G to A, U, or C, reduced cap-independent translation (Figure 5C) is consistent with this hypothesis.

Because (i) the TPAV PTE functioned in mammalian extract and cells, (ii) human eIF4E bound to the TPAV PTE in EMSA and (iii) protected the G-rich bulge in SHAPE footprinting, we deduce that TPAV PTE interacts with mammalian eIF4E in the same way it interacts with plant eIF4E to facilitate cap-independent translation. In contrast, mammalian eIF4E does not interact well with PEMV2 PTE which does not allow much cap-independent translation in mammalian systems. Thus, we speculate that the structures of the PEMV2 and other PTEs that do not function in mammalian translation systems differ from the TPAV PTE in a way that may not permit as much interaction with mammalian eIF4E. However, additional interactions besides with eIF4E may play a role in PTE function. We note that the PEMV2 PTE, which binds relatively weakly to eIF4E in EMSA (Figure 3A), inhibits translation relatively efficiently in wheat germ extract and RRL (Figure 1C and Figure 2C). This may be due to more efficient interaction when eIF4E is bound to eIF4G, which is the form in translation extracts, or due to possible functional interactions with other components of the translation extracts, which have not been ruled out. Also, because the TPAV PTE functions more efficiently than other PTEs in both plant and animal systems, it may not have a “mammalian-specific” motif, but may simply bind all eIF4Es (and possibly other factors), plant or animal, with higher affinity than do other PTEs.

The HCSRV PTE is also of interest because it functioned well in RRL and HeLa lysate, but only modestly in BHK cells. It is unique among PTEs in that it has five consecutive bases (six if a terminal U:G pair is included) of complementarity between the G- and C-domains, including a four base pair GGGG:CCCC tract, giving it a pseudoknot helix of greater stability than those of other PTEs (Figure S1) [32]. Before it was known to be part of a PTE, the HCSRV G-domain was found to be required for cap-independent translation in plants [53]. At the other extreme is the JINRV PTE which has no apparent Watson–Crick complementarity between the C- and G-domains, as there are no consecutive G’s in the G-domain [32]. This PTE functioned poorly in all assays except the RRLs which are the least cap-dependent (Figure 1 and Figure 2) [54]. However, the functionally weak PEMV2 PTE has a potentially stronger pseudoknot interaction (AGGG: CCCU) than that of the highly active TPAV PTE (GGG: CCC). Thus the efficiency of PTE function is not determined solely by predicted strength of pseudoknot. It is possible that our constructs lack all the sequences that contribute to base pairing between 3′ and 5′ ends required for optimal PTE function. Sequences in viral coding regions, which could enhance the long distance base pairing [55] were not tested. Thus, while we conclude that TPAV functions well in mammals, we cannot rule out that other PTEs may also do so in different constructs or conditions. However the EMSA assays show clearly that TPAV PTE has higher affinity than PEMV2 PTE for human eIF4E (Figure 3A, Table 1), supporting its superior translation enhancement activity. Future research is necessary to determine how the amino acid differences between human and wheat eIF4Es prevent human eIF4E from interacting (enough) with the non-mammalian-functioning PTEs, while interacting enough with the TPAV PTE to allow it to strongly stimulate translation.

### 3.2. Why No (known) 3′ CITEs in Animals?

Now that we have identified a 3′ CITE structure that functions efficiently in mammalian systems, we predict that such a PTE-like element may occur in uncapped mammalian viral RNAs or in capped host mRNAs to allow translation in conditions in which binding of eIF4E to the cap may be inhibited. Unfortunately, owing to the lack of sequence conservation, and the substantial secondary structural variability among known PTEs [32], it may be difficult to identify all PTE-like elements by computational methods.

Given the similarities of translation factors and translation mechanisms across kingdoms, it is perhaps surprising that there is little overlap in known sequences or structures of cap-independent translation elements, including IRESes, in plants and animals. In fact, in multiple experiments we have found that the BYDV BTE, which is a very powerful CITE in plants [47], has no stimulatory activity in mammalian systems (Figure 2, [48], and unpublished data). This may be because the BTE binds eIF4G rather than eIF4E, and requires a region of eIF4G that includes a potential RNA binding site unique to plant eIF4G [42]. However we have not ruled out the possibility that BTEs of other viruses may function in mammalian systems. In contrast, it is likely that the high structural similarity between plant and mammalian eIF4E (Figure 2A), is what allows *trans*-kingdom stimulation of translation by the TPAV PTE. On the RNA side of the interaction, we know of one example of structural similarity between viruses of different kingdoms that facilitates cap-independent translation. In this case, the large IRES in the 5′ UTR of *Triticum mosaic virus* (*Potyviridae*) contains the same Y_n_X_m_-AUG motif that is present in IRESes of picornaviruses [56], which infect vertebrates.

Although no 3′ CITEs have been found in animal viral or host mRNAs, structured regions in the 3′ UTR that control translation initiation at the 5′ end have been identified in the RNA genomes of hepatitis C [57,58,59], Sindbis [60], and Dengue [61,62,63] viruses, and in host mRNAs such as p53 mRNA [64]. Sindbis, Dengue and host mRNAs contain a 5′ cap. Thus, their translation is not cap-independent but they may interact with translation factors in additional ways beyond simply via the canonical eIF4E binding to the cap. One such noncanonical interaction takes place in the extremely conserved animal histone H4 mRNA, which contains an eIF4E-binding element within the coding region that regulates eIF4E recruitment to the mRNA, which is also capped [65].

The 3′ CITE-containing viruses are mostly in the *Tombusviridae* family and the closely related genus *Luteovirus* of the *Luteoviridae* [9,14]. By current knowledge, these viruses have no known modification at the 5′ end, and thus probably harbor a 5′ triphosphate. This is unlike any known animal positive strand RNA virus genome. RNAs with 5′ triphosphates induce innate immune response in mammals [66], but we are unaware of such an antiviral defense system in plants, which may explain why plant viruses can survive with a 5′ triphosphate. It is also possible that the population of 5′ to 3′ exonucleases in plants differs in such a way as to allow these genomes to last longer. However tombusvirid and luteoviral RNAs are clearly sensitive to 5′ exonucleolytic degradation, as many of them generate subgenomic RNAs by a mechanism that relies on host exonuclease degradation of the genomic RNA until an internal exonuclease-blocking structure (xrRNA) is reached, leaving an intact 5′-truncated subgenomic RNA [67,68,69].

Finally, we have proposed that CITEs exist in the 3′ UTR to facilitate switching off of upstream translation by the viral replicase as it moves from the 3′ to 5′ along the genomic RNA template [70,71]. Why 3′ CITEs are (so far) apparently absent on animal viral RNAs, preventing them from using this potential mechanism to switch off upstream translation remains open for speculation.

## 4. Materials and Methods

### 4.1. eIF4E Structure Alignment

eIF4E crystal structures from human (representing mammalian eIF4E) and wheat were obtained from Protein Data Bank (accession numbers 1IPC and 2IDV, respectively), overlaid using the “iterative Magic fit” tool, and RMS values calculated using Swiss PDB Viewer v. 4.03 [72]. Cartoon renderings were produced using Persistence of Vision Raytracer (Pov-Ray) v. 3.62 (PoVP Ltd. 2004).

### 4.2. Plasmid Constructs and RNA Synthesis

The luciferase constructs used in translation assays consisting of the firefly luciferase gene (luc2, Promega) flanked by the indicated viral genomic 5′ and 3′ UTRs [32] were linearized with Sma I and transcribed using the MEGAscript kit (Ambion). CAluc, which is a capped transcript containing nonviral UTRs described previously [73], was linearized with EcoICRI, transcribed with the MEGAscript kit (Ambion) and post-transcriptionally capped using the T7 mScript Standard mRNA Production System (Cell Script).

For *trans*-inhibition and structure probing experiments, the PTE sequences from the indicated viruses present in the universal SHAPE cassette [15,32] were linearized with either HpaI (giving only PTE transcript for *trans*-inhibition studies) or SmaI (which adds a 3′-terminal extension on the PTE providing a primer binding site for SHAPE experiments) and transcribed in vitro using the MEGAshortscript kit (Ambion). All transcripts were purified by phenol/chloroform extraction and ethanol precipitation. RNA concentrations were determined spectrophotometrically and integrity was verified by 0.8% agarose gel electrophoresis.

### 4.3. Recombinant Protein Expression

His-tagged wheat eIF4E in pET23d vector was introduced into *E. coli* (BL21 cells) and expression was induced at OD 600 nm = 0.8, with 100 mM IPTG. After 4 h of induction, cells were harvested from 1 L of culture by centrifugation at 10,000× *g* for 10 min. The cells were frozen at −80 °C for at least 1 h and sonicated 12 times for 30 s each with 2 min cooling on ice in binding buffer [25 mM HEPES–KOH at pH 7.6, 100 mM KCl, 2 mM MgCl_2_, 10% glycerol plus 0.1 mM phenylmethyl–sulphonyl fluoride, 0.1% Soybean trypsin inhibitor and 1 tablet/10 mL of Complete protease inhibitor cocktail, EDTA-free (Roche)]. The homogenate from 1 L of cells was centrifuged at 38,000× *g* for 20 min at 4 °C and supernatant was applied to 1 mL of Ni-NTA Superflow Cartridge (Qiagen). The cartridge was washed with 10 volumes of binding buffer plus 10mM imidazole and then with 10 volumes of binding buffer plus 20 mM imidazole. The his-tagged proteins were eluted with 250 mM imidazole in the same buffer. Human eIF4E used in structure probing experiments was a kind gift of Franck Martin, Institut de Biologie Moléculaire et Cellulaire Strasbourg, France.

### 4.4. Translation in Plant Systems

In vitro translation was set up using 2 nM of capped CAluc or uncapped viral luciferase reporter transcripts in either wheat germ extract as described by the manufacturer (Promega) or BY-2 cell extracts as described by [74] with some modifications [42]. Translation reactions were incubated for 1 h at 22 °C and the luciferase activity was measured by addition of 2 µL of the translation reaction to 40 µL of the Luciferase Assay Reagent (Promega). Relative light units were obtained using a GloMaxTM20/20 luminometer. Each construct was tested in triplicate in each experiment, and in at least three independent experiments.

For translation in vivo (protoplasts) 2 nM of capped CAluc or uncapped viral luciferase reporter transcript was mixed with 0.2 nM of capped and poly(A)60-tailed *Renilla*-luciferase reporter mRNA [75] and electroporated into approximately 1 million oat (*Avena sativa* cv. Stout) protoplasts that were prepared as described previously [76]. After 4 h incubation at room temperature, protoplasts were harvested and lysed in Passive Lysis Buffer (Promega). Both *Renilla* and firefly luciferase activities were measured using Dual-Luciferase reporter assay system (Promega). To account for variations between experiments in electroporation efficiency and protoplast quality, the firefly luciferase activities were normalized to the *Renilla* luciferase activity.

*Trans-inhibition of translation.* Non-saturating amounts (2 nM) of uncapped transcript containing 5′ and 3′ UTRs of PEMV2 flanking firefly luciferase ORF, or capped CAluc transcript, pre-mixed with 200 nM of the designated viral PTEs were translated in wge (Promega) for 1 h at 22 °C as described [16,32]. The luciferase activity was measured by addition of 2 µL of the translation reaction to 40 µL of the Luciferase Assay Reagent (Promega) followed by measurement of relative light units in a GloMaxTM20/20 luminometer. The relative activity obtained from P2lucP2 or CAluc in the absence of inhibitor was defined as 100%.

### 4.5. Translation in the Mammalian Systems

T7-BHK cells were cultured as described previously [52,77]. Briefly, cell growth media was prepared by supplementing Glasgow Minimal Essential Media (GMEM) with amino acids, 10% fetal bovine serum (FBS), and penicillin-streptomycin. G418 (geneticin), was added every other passage at a concentration of 1 mg/mL to maintain the T7 expression construct. The cells were grown at 37 °C with 7% atmospheric CO_2_, and passaged when they reached approximately 80% confluency.

Transfection was performed using Invitrogen Lipofectamine Transfection Reagent using the manufacturer’s protocol on cells that had been seeded onto 6-well plates at a density of 2 × 10^5^ cells/well and allowed to reach approximately 80% confluency. Significant cell death was not observed upon transfection. Twenty-four hours after transfection, the cells were placed on ice, removed from the plate by scraping, and lysed using Lysis Buffer (Promega). Lysate was diluted 1:4 in Lysis Buffer, then 20 µL was added 100 µL of Luciferase Assay Reagent (Promega), and quantified with the GLO-MAX luminometer, or frozen and stored at −80 °C until quantification.

### 4.6. Electrophoretic Mobility Shift Assay (EMSA)

Prior to electrophoresis, 2000 cpm ^32^P-labeled RNA (20 fmol) was incubated with indicated concentrations of purified eIF4E in binding buffer: 10 mM HEPES pH 7.5, 20 mM KCl, 1 mM dithiothreitol, 3 mM MgCl_2_, 1 µg/µL tRNA, 0.5 µg/µL bovine serum albumen, 1 unit/µL RNaseOUT™ Recombinant Ribonuclease Inhibitor (Invitrogen), 5% glycerol, in a total volume of 10 µL for each sample. After 25 min on ice, 3 µL 50% glycerol with bromophenol blue was added, and the RNA-protein mixture was loaded on a 5% polyacrylamide (acrylamide:bis-acrylamide 19:1), Tris-borate/EDTA gel. After electrophoresis at 110 V for 45–60 min (4 °C), gel was dried and exposed to phosphorimager screen overnight. After scanning the screen in a Bio-Rad PhosphorImager, data were analysed using Quantity One software (Bio-Rad) to calculate the apparent K_d_s. Shifted (bound) and unshifted (unbound) bands were each quantitated from three separate experiments. Standard error was calculated and significance of pairwise comparisons of human vs wheat eIF4E, TPAV versus TPAVm2 PTE, and capped versus uncapped RNAs was determined using Student’s t-test.

### 4.7. RNA Structure Probing and Footprinting

Chemical and enzymatic RNA structure probing was performed as described previously [42]. Briefly, 500 ng of refolded RNA alone or pre-incubated for 10 minutes with indicated proteins was treated with 10% (v/v) of benzoyl cyanide (Sigma-Aldrich) and incubated for 30 s at 22 °C. As a control, RNA refolded in the presence of 3 mM Mg^2+^ was treated with 10% (v/v) of DMSO in place of chemical reagent. RNA was then purified by phenol–chloroform extraction and ethanol precipitation. Reactions were resolved on an 8% denaturing polyacrylamide gel and dried following primer extension. Dried gels were exposed to a storage phosphor screen as described previously [32,42]. Each experiment was repeated at least three times.

## Figures and Tables

**Table 1:** Dissociation constants (Kd) for eIF4E binding to PTE RNA in electrophoretic mobility shift assaysa.

**Kd (nM)**						
	**TPAV PTE**		**TPAV m2 PTE**		**PEMV RNA2 PTE**	
eIF4Es	capped	uncapped	capped	uncapped	capped	uncapped
human	160 ± 1	838 ± 124	253 ± 45	6362 ± 1283	285 ± 33	2524 ± 435
wheat	31 ± 7	65 ± 14	26 ± 2	463 ± 185	41 ± 25	110 ± 38
**Fold Kd human/wheat eIF4E**						
	**TPAV PTE**		**TPAV m2 PTE**		**PEMV RNA2 PTE**	
	capped	uncapped	capped	uncapped	capped	uncapped
	5.16 ***	12.89 **	9.73 **	13.74 **	6.95 ***	22.94 **
**Fold Kd uncapped/capped RNA**						
	**TPAV PTE**		**TPAV m2 PTE**		**PEMV RNA2 PTE**	
	human	wheat	human	wheat	human	wheat
	5.24 **	2.10 *	25.15 **	17.81 *	8.86 **	2.68 *
**Fold Kd TPAVm2/TPAV RNA**						
	**human eIF4E**		**wheat eIF4E**			
	capped	uncapped	capped	uncapped		
	1.58 *	7.59 **	0.84 ^n.s^	7.12 *		

Student’s t-test P values: * 0.01 < P < 0.05, ** 0.001 < P < 0.01, *** P < 0.001, n.s. not significant, P > 0.05; a All samples measured in three separate experiments, quantified by phosphorimager analysis.

## Supplementary Materials

The following are available online at https://www.mdpi.com/2076-0817/8/1/28/s1, Supplementary Figure S1: Secondary structures of PTEs determined by Wang et al., Supplementary Figure S2: Determination of the structural parameters of TPAV-human eIF4E interaction.
