# Supplementary material for: The 3′ Untranslated Region of a Plant Viral RNA Directs Efficient Cap-Independent Translation in Plant and Mammalian Systems
Source: Pathogens. 2019 Feb 28;8(1):28. doi: 10.3390/pathogens8010028 (PMC6471432; doi:10.3390/pathogens8010028)
Supplement: Supplementary file 1 [file pathogens-08-00028-s001.pdf]

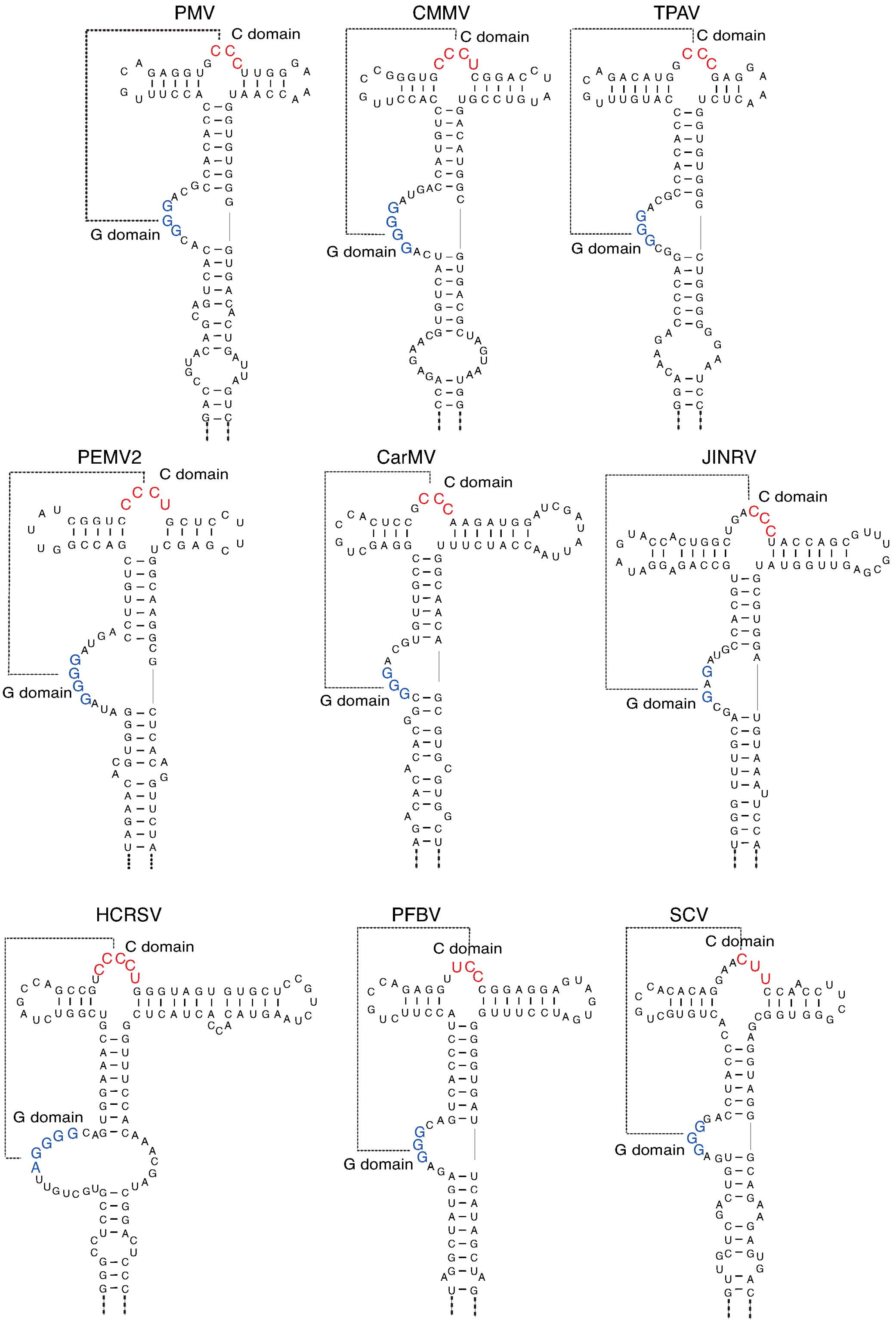

Supplementary Figure S1. Secondary structures of PTEs determined by Wang et al. [32]. Bases predicted to base pair in a pseudoknot interaction (dashed line) are indicated in enlarged red (C domain) and blue (G domain) text. PMV: panicum mosaic virus; CMMV: cocksfoot mild mosaic virus; CarMV: carnation mottle virus; PFBV: Pelargonium flower break virus; JINRV Japanese iris necrotic ringspot virus; SCV: saguaro cactus virus.

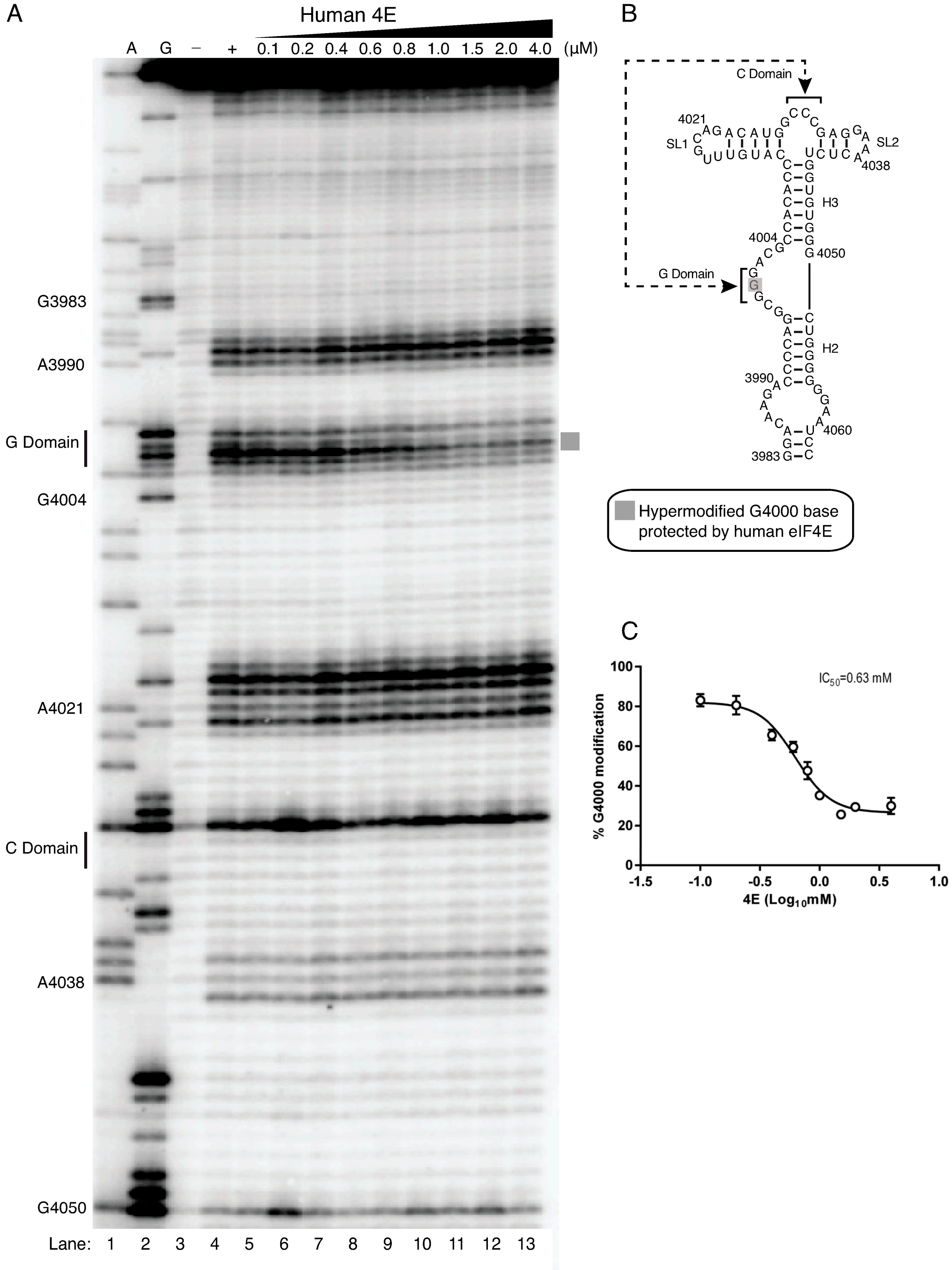

Supplementary Figure S2. Determination of the structural parameters of TPAV-human eIF4E interaction. (A) BzCN footprinting of the TPAV RNA in the presence of increasing concentrations of human eIF4E. A and G are dideoxy sequencing lanes and unmodified RNA (- lane) shows background RNA hydrolysis. (B) TPAV PTE secondary structure with highlighted G4000 nucleotide that shows reduced modification by BzCN in presence of human eIF4E. (C) Quantification of SHAPE reactivity of TPAV PTE G4000 nucleotide in presence of increasing quantities of human eIF4E. Each point represents an average from at least three independent experiments, and bars represent standard error. Data were fitted using GraphPad software. **Methods: RNA structure probing and footprinting**. Chemical and enzymatic RNA structure probing was performed as described previously [42]. Briefly, 500 ng of refolded RNA alone or pre-incubated for 10 min with indicated proteins was treated with 2 10% (v/v) of benzoyl cyanide (Sigma-Aldrich) and incubated for 30 s at 22° C. As a control, RNA

refolded in the presence of 3 mM Mg2+ was treated with 10% (v/v) of DMSO in absence of BzCN. RNA was then purified by phenol–chloroform extraction and ethanol precipitation. Reactions were resolved on an 8% denaturing polyacrylamide gel and dried following primer extension. Dried gels were exposed to a storage phosphor screen as described previously [32,42]. The experiment was repeated at least three times. To plot the decrease in SHAPE reactivity at G4000 in the TPAV PTE upon addition of human eIF4E (Supplementary Figure S2C), we quantitated the total RNA signal for each lane separately using a Typhoon Phosphorimager. First, the percent of total RNA in each lane (a given eIF4E concentration) that is represented by G4000 signal was calculated. This was then used to calculate the percent G4000 signal relative to that at 0 mM eIF4E. These values were plotted in GraphPad against Log10 of 4E concentrations to calculate the IC50: the concentration of eIF4E at which G4000 is 50% modified by BzCN relative to modification in the absence of eIF4E.
